# Health(care) in the Crisis: Reflections in Science and Society on Opioid Addiction

**DOI:** 10.3390/ijerph18010341

**Published:** 2021-01-05

**Authors:** Roxana Damiescu, Mita Banerjee, David Y. W. Lee, Norbert W. Paul, Thomas Efferth

**Affiliations:** 1 Department of Pharmaceutical Biology, Institute of Pharmaceutical and Biomedical Sciences, Johannes Gutenberg University, 55128 Mainz, Germany; r.damiescu@uni-mainz.de; 2 Department of English and Linguistics, Obama Institute for Transnational American Studies, Johannes Gutenberg University, 55128 Mainz, Germany; mita.banerjee@uni-mainz.de; 3 McLean Hospital, Harvard Medical School, Boston, MA 02478, USA; dlee@mclean.harvard.edu; 4 Institute for History, Philosophy and Ethics of Medicine, Johannes Gutenberg University Medical Center, 55128 Mainz, Germany; npaul@uni-mainz.de

**Keywords:** OxyContin, opioid abuse, chronic pain, patient narratives

## Abstract

Opioid abuse and misuse have led to an epidemic which is currently spreading worldwide. Since the number of opioid overdoses is still increasing, it is becoming obvious that current rather unsystematic approaches to tackle this health problem are not effective. This review suggests that fighting the opioid epidemic requires a structured public health approach. Therefore, it is important to consider not only scientific and biomedical perspectives, but societal implications and the lived experience of groups at risk as well. Hence, this review evaluates the risk factors associated with opioid overdoses and investigates the rates of chronic opioid misuse, particularly in the context of chronic pain as well as post-surgery treatments, as the entrance of opioids in people’s lives. Linking pharmaceutical biology to narrative analysis is essential to understand the modulations of the usual themes of addiction and abuse present in the opioid crisis. This paper shows that patient narratives can be an important resource in understanding the complexity of opioid abuse and addiction. In particular, the relationship between chronic pain and social inequality must be considered. The main goal of this review is to demonstrate how a deeper transdisciplinary-enriched understanding can lead to more precise strategies of prevention or treatment of opioid abuse.

## 1. Introduction

The current coronavirus crisis has brought into sharp relief that while no one is immune to the virus, an epidemic may affect groups to different degrees. Thus, the COVID-19 pandemic has shown that socially and economically disenfranchised groups are particularly at risk in an epidemic. Dramatically, the U.S. currently seems to be in the grip not only of the COVID-19 epidemic, but also of another major crisis: the so-called opioid epidemic, which is now also spilling into many areas of the world. This paper demonstrates that in order to develop effective solutions to this crisis, a multidisciplinary approach is necessary, which links the life sciences and the humanities. For an in-depth understanding of the trends in opioid use and abuse, it is essential to understand a complex network of not only doctors, pharmaceutical companies, patients and institutions, but also society and social factors at large. This becomes even more evident, if studies indicate that on a therapeutic level, management of pain could or should effectively be combined with cognitive behavioral therapy [1,2]. For such therapeutic strategies, in turn, it may be crucial to understand not only the individual factors, but also cultural and societal factors which may lead to opioid addiction. As the American Psychological Association (APA) suggests, cognitive behavioral therapy (CBT) might contribute to strategies for coping with addiction, which are better embedded in a reconceptualization of the affected individuals [3]. While it is not surprising that the APA walks down the paved roads of dealing with addiction (like in alcohol or heroin abuse), the strategy sheds some light on the lack of preventive strategies. First, this paper combines approaches from pharmaceutical biology and the humanities in order to foster a multidisciplinary approach to combatting the opioid crisis by a more preventive approach. Second, we would like to recommend routes for further research where this multidisciplinary approach could be applied to complement the health impact assessment of opioid addiction with health needs assessment. We argue that an assessment of institutional and local measures to tackle the opioid crisis is urgently needed, in order to investigate which preventive measures might be effective and thus become a venture point for the policy making which is urgently needed to effectuate a public health approach to the opioid crisis locally, nationally and globally.

This review will first provide an overview of the opioid crisis and its inception, focusing specifically on OxyContin™ as a key example. Secondly, it will explore factors often associated with opioid overdose and analyze the necessity of opioid chronic pain management, also looking at post-surgery pain management as an entry point for opioids in the lives of patients. It will then examine the mechanisms behind an opioid addiction from the perspective of pharmaceutical biology. The discussion will subsequently lead to a patient perspective, exploring the role of patient compliance and the cultural framing of opioid addiction. In the final part of this paper, current avenues for treatment will be examined. In the conclusion, we explore the potential advantages and challenges of an interdisciplinary approach, which combines the life sciences and the humanities [4] and we suggest that without such interdisciplinarity, we may fail to grasp the current opioid crisis in all its complexity. Finally, while this article discusses the opioid crisis in the U.S. specifically, it also has worldwide implications for the use of opioid-based pain medication. What are the factors which might lead to such a global opioid epidemic? Crucially, through its interdisciplinary approach, this review thus seeks to contribute to preventing a further spread of the epidemic, both on the level of the U.S. and on the global level. A recent published report has argued that it is important to tackle the opioid crisis by “going back to its roots” [5]. Through understanding the mechanisms which led to the U.S. opioid epidemic, we may be able to stop it from flooding Europe as well. At the same time, it demonstrates that most discussions so far have failed to take cultural and social perspectives sufficiently into account. This paper, however, argues that the “root” of the problem of the opioid crisis may not only be medical, but it may also be a cultural and a social problem. For this reason, the following discussion links the methodologies of the life sciences and the humanities in explaining the opioid epidemic in its current form. All this, however, needs to be rooted in a sound understanding of the role of pain and pain management as it is practiced *legis arte* today.

The World Health Organization (WHO), together with the International Association for the Study of Pain, have recognized proper pain management as a fundamental human right [6]. Nonetheless, adequate pain management, particularly if it comes to chronic pain, has been a challenge both for doctors and for patients, who have been facing the problem of insufficient pain management for years (as the goal is to reduce pain with minimum side effects) and the ubiquitous presence of opioids with little thought on adoption, addiction and abuse in the last decade. Even after decades of research in pain management, opioids still remain the most prescribed drugs for treating postoperative pain [7,8,9]. Historically, the isolation of morphine from the opium poppy in 1805 by Friedrich W. A. Serturner was a turning point for pain management in medicine. After more than 200 years, morphine is still one of the most prescribed opioids and is used as a standard to compare the potency of other opioids [10]. However, because of its pharmacokinetic profile, scientists have tried to synthesize stronger and more effective compounds. If administered orally, morphine undergoes a significant first-pass effect, as it metabolizes through the liver to morphine-6-glucuronide, a more potent analgesic; it has a half-life of 2–3 h, requiring a new administration approximately every 4 h [11]. Following repeated use, it leads to tolerance and physical dependence [11]. Therefore, scientists have searched for more potent opioids with a better bioavailability and as such the release of OxyContin™ in 1996 by Purdue Pharma in USA seemed like a promising alternative. However, even then multiple studies concluded that there is no comparable advantage in comparison to other oxycodone preparations, besides a reduced dose frequency [12]. In this light, one key questions needs to be answered: how did opioids become America’s most used and misused drugs?

## 2. How Did Opioids Become America’s Most Used and Misused Drugs?

The Food and Drug Administration (FDA) is responsible under the Food, Drug and Cosmetics Act to regulate the advertising and promotion of prescription and noncontrolled drugs in order to assure their truthfulness and have a positive impact on public health. The U.S., together with New Zeeland, remain the only two countries in the world which allow direct-to-consumer advertising of prescription drugs. Back in 1996, shortly after introducing OxyContin™ on the market, Purdue Pharma started a highly aggressive marketing and promotion campaign without submitting promotional videos to the FDA to be reviewed [13]. Purdue Pharma conducted multiple conferences on pain management, which were attended by over 5000 physicians, pharmacists and nurses, who were then recruited as Purdue’s speakers [13]. Not only did Purdue Pharma target primary care physicians, but they also offered branded promotional items to healthcare professionals and various coupons for OxyContin™ for new patients [13]. During the same time pain was described as “the fifth vital sign” and the problem of more than seldom undertreated pain attracted the attention of healthcare providers, who then decided to improve the treatment guidelines of pain management [14,15]. This led to a more liberal use of opioids in the treatment of pain. Purdue promoted the use of Oxycontin™ in non-cancer-related pain and assured that the risk of addiction was low due to its formulation as a controlled-release tablet [12]. With the intent to improve pain management and patient outcomes, doctors started overprescribing strong opioids, creating a slippery slope. By 2005, through the liberalization of prescription opioids, nationwide there was an increase in opioid prescriptions and drug abuse [16,17]. Oxycodone became the most abused prescribed opioid in the USA and led to more deaths than heroin [18]. In 2010, Purdue Pharma released an abuse-deterrent formulation (ADF) for oxycodone, making it harder to crush or dissolve, and in 2013 the FDA decided the prior formulation should be removed from the market for safety reasons. Several studies have examined the efficacy of the abuse-deterrent formulation and have concluded that it is associated with a decline in the abuse of oxycodone [19,20,21,22]. However, one study, which also included patients misusing prescription opioids or heroin, highlighted the limited effectiveness of the abuse-deterrent formulation, as the level of residual abuse has remained stable despite the new formulation [19]. Although the ADF has reduced the abuse of oxycodone, the active compounds in these formulations preserve their euphoric effects and strong side effects, including overdose potential. As such, the search for new analgesics continued and a new approach targeted the peripheral opioid receptors using hydrophilic substances and polyglycerol-based nanocarriers; this strategy has shown promising results [23]. Other strategies include increasing endogenous opioid mechanisms, use of allosteric modulators, bivalent ligands and bivalent signaling [23].

Even though great advancements have been made in the practice of surgery, as techniques have become more precise and less invasive [24] in order to improve the outcomes, opioids are still the first choice for postoperative pain management [25,26]. In the attempt to improve recovery and quality of life, as well as reduce hospital stays, physicians started prescribing more opioids, so that up to 80% of patients in the USA receive strong opioids after a surgical procedure [9].

Due to increased morbidity associated with opioid overdose, various studies have started analyzing the factors influencing the opioid prescribing patterns in opioid-naive patients. Opioid-naive patients are defined as patients with no prescription for any opioids in the last 6 to 12 months prior to their first opioid prescription and with no history of substance abuse [9,27,28]. The trends in opioid prescriptions in opioid-naive patients after low-risk surgical procedures (e.g., carpal tunnel release) have been analyzed for a time period from 2004 to 2012. The study evaluated the proportion of patients filling an opioid prescription (especially oxycodone/acetaminophen or hydrocodone/acetaminophen) within 7 days after a procedure. Seventy percent of them filled an opioid prescription within a week. Additionally, there was an increase in opioid dose of 18% for all procedures [9]. A more complex investigation associated the characteristics of the initial opioid use with pain etiology. The most common indications for opioid prescription are chronic non-cancer pain, surgery, trauma and burns. The authors concluded that patients initiated with tramadol, long-acting opioids or doses over 90 morphine milligram equivalents were more likely to continue opioid use [28] Additionally, multiple studies have characterized the duration of the prescription and high opioid dose as strong predictors of the likelihood of long-term use [27,28,29,30]. Other risk factors are continued use of other medicines (benzodiazepines, muscle relaxants), history of alcohol, tobacco or drug abuse, lower socioeconomic status and psychiatric disorders [28,31,32] which will, unfortunately, most likely increase as a consequence of the ongoing COVID-19 pandemic and the related anxiety and social deprivation. As numbers of opioid overdose deaths continue to grow in the U.S., Centers for Disease Control and Prevention (CDC) discovered that in 63% of cases there is also another drug involved. Both alcohol and CNS depressants are known to cause respiratory depression, therefore mixing them can have fatal effects. A recent study gathered data from the last 20 years and confirmed that co-involvement of alcohol or benzodiazepines in opioid overdose deaths (OOD) is common and the prevalence rate is increasing for alcohol from 12.4% (1999) to 14.7% (2017) and for benzodiazepines from 8.7% (1999) to 21.0% (2017), more and more replacing the former culturally accepted (but now increasingly unacceptable drug), alcohol, with a “medication”. Alcohol is still more commonly used by men [33], while benzodiazepines are more frequently used by women [33]. The proportions of co-involved alcohol and benzodiazepines vary depending on the opioid subtype (prescription opioid vs. illicitly manufactured), as illicitly manufactured fentanyl has been responsible for almost half of the opioid overdose deaths since 2015 [33]. These results should be considered by physicians when prescribing both opioids and benzodiazepines and should be integrated in public health communication since many patients, at first, seem to not be fully informed of potential risks. The prescription rates of benzodiazepines have also increased substantially in the last decades, as they have become the most prescribed sedatives [34]. In 2016, the CDC published a guideline with recommendations for prescribing opioids for chronic pain with the intent to improve the effectiveness of treatment and reduce adverse events and risks. In the guidelines, CDC recommends starting an opioid therapy for chronic pain only if the benefits outweigh the risks and the therapy should be combined with nonopioid (NSAIDs, anticonvulsants and antidepressants) and nonpharmacologic therapy (CBT, exercise therapy). Clinicians should determine realistic goals, discuss the risks with the patients and only continue therapy if significant improvement appears. Additionally, opioid therapy for chronic pain should start with the lowest effective dose and immediate-release opioids are preferred to extended-release. Thus, an evaluation of the therapy benefits should be done within the first 4 weeks after starting the treatment and then every three months, which is in many Western countries the threshold for developing chronic pain. Generally, the CDC recommends avoiding the concurrent prescription of opioids and benzodiazepines, as the risks are higher than the benefits. Last but not least, a patient’s medical history should be reviewed, and urine drug tests should be done annually, in the case of long-term treatment. If there are any risk factors involved, clinicians must establish strategies to minimize the risks [35]. To improve the knowledge of healthcare providers, avoid overprescribing opioids and help them apply these guidelines, the CDC now offers different interactive online training programs targeting professional groups.

Since opioids are frequently prescribed after both minor and major surgeries, scientists have wanted to determine how often after a surgical procedure patients become chronic opioid users and which associated risk factors (alcohol, tobacco, depression) can influence the outcome [36,37]. Although chronic opioid use is common after surgery, the rates were low [36,37,38]. A report from 2016 analyzed the risk of chronic opioid use in opioid-naive patients after having 1 of the 11 most common surgical procedures, compared to nonsurgical patients. Excluding the first 90 days post-surgery, surgical patients were considered chronic opioid users if during the first-year post-surgery, they had a supply of opioids for over 120 days or filled over 10 prescriptions. In the case of the nonsurgical patients, a “surgery date” was assigned randomly, and chronic users were defined by using the same criteria. Even though two of the surgical procedures are often associated with pain (total knee arthroplasty, total hip arthroplasty), the authors suggest different pre- and postoperative techniques for pain management. The study confirmed the risk of chronic opioid use during the postoperative period and also highlighted the potential of different risk factors (history of drug or alcohol abuse, use of antidepressants and benzodiazepines) in the patient outcomes [36]. These findings were also confirmed by other authors, who observed that the risk of chronic opioid use in opioid-naive patients following a minor or major surgical procedure is increased, and it is especially associated with other risk factors (alcohol and drug abuse, use of benzodiazepines, antidepressants) and preoperative pain disorders (arthritis, back pain) [37]. These data describe the influence of psychiatric conditions in chronic opioid use. To increase awareness regarding the risk of chronic opioid use, a new analysis focused on both opioid-naïve and opioid non-naïve patients receiving opioids in the perioperative period. For both group of patients, it was confirmed that, if patients received over 450 Morphine Milligram Equivalent (MME)and a high amount of opioid medication, they were more likely to become chronic opioid users [38]. Additionally, various guidelines were proposed with the purpose of reducing opioid prescriptions following surgical procedures. The first study describes that from 85% of patients who received an opioid prescription at discharge on postoperative day 1, only 38% of the prescribed opioids were taken. The number of opioid pills taken the day before discharge were correlated with the number of opioid pills taken afterwards. As such, the guideline proposes the following: no opioid prescription if the patient does not need any opioids the day prior discharge; if the patient takes 1–3 opioid pills, then they receive an opioid prescription for 15 pills and if the patients require more than 4 opioid pills, they obtain a prescription for 30 opioid pills [39]. Additionally, the same group prepared another guideline for surgeons to decrease postoperative opioid prescriptions. Surgeons were recommended to prescribe NSAIDs or acetaminophen as first choice therapy. As such, the number of opioid pills decreased by over 50% and 85% of patients received acetaminophen or a NSAID and did not require opioids afterwards [40]. These studies demonstrate that although appropriate postoperative pain management remains a complex matter, surgeons often overprescribe opioids.

In the last decades opioid therapy has been frequently prescribed for chronic pain and as an undesired outcome the number of opioid overdoses has increased. A randomized clinical trial supported the efficacy of short-term (12 weeks) opioid therapy in treating chronic pain [35]. However, their efficiency was not higher compared to nonopioid therapy [35]. Regarding benefits of long-term opioid therapy, evidence still remain insufficient. Thus, various studies have concluded that higher doses of opioids are often associated with an increased risk of harm, opioid abuse and overdose [41,42,43,44]. Although the risks are well known, reducing the dose was easier said than done for patients, who had already taken high doses of opioids for a longer period. A study from 2014 evaluated, using high-dose chronic opioid users, if the recognized benefits and harms can be used as predictors of high-dose use after one year. A majority of patients (74%) reported at least one side effect and many of them reported concerns and problems associated with opioid use. Even though almost half of the patients expressed the wish to reduce or stop opioid therapy, 80% of them continued to use high doses after one year [45]. Other studies confirmed that despite the fact that chronic patients report multiple side effects and problems related to high doses of opioids, the rates of opioid reduction were still low [41,44,46]. These results reinforce how difficult it is for chronic opioid users to reduce doses even though it could improve their quality of life. Although a decrease in opioid prescribing already started in 2012 after the CDC guidelines were published [35], opioid prescriptions decreased at a higher rate [47]. On the other hand, this was also associated with increased illicit opioid use (heroin and illicitly manufactured fentanyl) and overdose deaths [48], as people already dependent on the opioid therapy suddenly stopped receiving their medication. A retrospective cohort study determined that patients, who had treatment discontinued and those whose doses were increased were more likely to use heroin and illicit fentanyl [49]. All in all, the available data confirm the complexity of the opioid problem and that the abrupt reduction in opioid prescriptions can produce more harm than good. Hence, it is an ethical postulate that clinicians should carefully evaluate and discuss with the patients the best measures that should be taken to reduce or cease opioid therapy without negatively affecting the quality of life. To help safely reduce opioid doses in chronic patients, various risk mitigation initiatives have been started. These initiatives have proven to be very efficient, as both high-risk patients (those with history of mental or substance use disorder) as well as low-risk patients managed to reduce their daily doses of opioids [50]. Beyond clinical practice and behavioral factors, opioid addiction is also deeply rooted in pharmacological and neurobiological mechanisms.

Although the opioid crisis has mainly affected the U.S. and Canada, a report showed that opioid prescription rates have nearly doubled in Europe during the past two decades and there are approximately 1.3 million high-risk opioid users, and the countries with the highest consumption of opioids are Germany, the UK, France, Spain, Italy and the Netherlands [51]. In comparison to the U.S., the number of opioid-related overdose deaths and hospitalizations is still low, and while in the U.S. most overdose deaths are caused by fentanyl and other synthetic opioids, in Europe heroin is attributed to most fatal overdoses [51]. Additionally, in Europe the Access To Opioid Medication in Europe (ATOME) project has provided information about opioid medication in legal, societal and policy contexts in order to help to implement various European policies [52].

A recent review has analyzed the trends in opioid prescription practices in Germany, as the country is the second largest opioid consumer in Europe, and although opioids are mostly being prescribed to treat chronic, non-cancer pain, the study concluded that currently there are no signs of an opioid epidemic [53]. New studies provide information on a national level regarding the impact of opioid misuse in different European countries such as France, the Netherlands and the UK.

As the number of opioid prescriptions has drastically increased during the past two decades, an investigation from the Netherlands reported that proxies for opioid misuse augmented, the number of opioid prescriptions, substitution therapies and opioid-related deaths doubled and opioid-related hospital admissions tripled [54]. An analysis from France from 2004 to 2017 described a significant increase in oxycodone use (+1950%), opioid-related hospitalization (+167%, 2000–2017) and opioid overdose deaths (+146%, 2000–2015) [55]. Furthermore, in the UK, the number of opioid prescriptions quadrupled, and from 1993 to 2017 opioid overdose deaths rose drastically [54]. All three studies highlight the importance of safe opioid prescribing guidelines and adequate monitoring of opioid use in the respective countries.

## 3. What Are the Mechanisms Behind Opioid Addiction?

In order to better understand how the use of opioids affects the brain, leading to tolerance and dependence in chronic users, it is best to first take a look at their mechanism of action. The opioid receptor family includes μ—(mu), δ—(delta) and κ—(kappa) receptors and the more recently discovered nociception/orphanin FQ receptor, all belonging to the G-protein-coupled receptor class [11]. Plus, depending on the produced response, opioids can classically be categorized as full agonists (morphine, oxycodone, methadone), partial agonists (buprenorphine, tramadol, nalbuphine) or antagonists (naloxone, naltrexone) [11]. However, recent developments have identified new classes: mixed MOR/DOR agonists, MOR/NOR agonists (cebranopadol), as well as biased agonists (oliceridine, PZM21, mitragynine pseudoindoxyl, SR-17018) [56]. The concept of biased signaling seemed to be a promising therapeutic strategy, as the arrestin pathway promotes side effects, while G-protein signaling induces analgesic effects. However, preclinical and clinical trials have demonstrated that biased agonists cause the same side effects as opioids [23]. On a cellular level, an opioid agonist binds to the G-protein-coupled receptor causing the α subunit to replace guanosine diphosphate (GDP) (inactive form) with intracellular guanosine triphosphate (GTP) (active form) and, hence, inhibits the adenylyl cyclase. This leads to a reduced production of cyclic adenosine monophosphate (cAMP) production. Plus, the α-GTP complex also interacts with the ion channels, producing hyperpolarization of the cells by activating the K^+^ channels and decreasing neurotransmitter release by inhibiting Ca^2+^ channels [23,57].

Different kinases phosphorylate the opioid receptors and promote arrestin recruitment leading to receptor desensitization and internalization. Arrestin, as a scaffolding protein, can be involved in both recycling and recovery of dephosphorylated opioid receptors or degradation by lysosomes [23].

Additionally, the activation of the opioid receptors can cause different effects, besides analgesia, depending on the location of the receptor. Activation of μ-opioid receptors produces analgesia, respiratory depression, sedation, reduced gastric motility, nausea, vomiting and miosis. The binding of agonists to δ-opioid receptors leads to spinal and supraspinal analgesia and reduced gastric motility, and the activation of κ-opioid receptors causes spinal analgesia, dysphoria and diuresis [57]. Due to their lipophilicity, opioids can pass through the blood–brain barrier and enter the central nervous system (CNS). In the brain, opioids attach to the μ-opioid receptors and cause the analgesic effect. In this manner, opioids also activate other brain processes like the mesolimbic reward system which signals the ventral tegmental area (VTA) to release dopamine and stimulate dopamine 1 (D_1_) receptors in the nucleus accumbens (NAc). The fast release of dopamine activates the reward processes, causing feelings of pleasure. Opioids are not the only drugs that increase dopamine levels in the NAc. Other substances of abuse achieve similar effects through various mechanisms. Cocaine blocks dopamine transporters and inhibits the removal of dopamine from the synaptic space, leading to enhanced dopamine levels. Alcohol increases the levels of γ-aminobutyric acid (GABA) neurotransmitter in the brain, which leads to accumulation of dopamine in the VTA [58]. Additionally, another study has associated a maximum drug reward with quickly increased dopamine levels and the binding of dopamine to both D_1_ and D_2_ receptors. Through brain imaging of humans, it was observed that the brain reward mechanism is activated by drugs, if dopamine increases over a short period of time (<10 min), while the slow release of dopamine over a longer period of time (1 h) did not [59]. A continued consumption of these substances of abuse (opioids, cocaine) triggers the constant release of dopamine in the NAc, which leads to the craving of the drug. Nevertheless, the mesolimbic rewards system is also regulated by endogenous opioids. The endogenous opioid system influences hedonic responses and the modifications occurring with repeated drug abuse and activates κ-receptors, blocking dopamine release in NAc. Chronic drug abuse alters the physiological functions of the brain and triggers systems to restore the natural balance in the brain. As such, repeated and excessive release of dopamine in the NAc eventually leads to the activation of auto-receptors to inhibit dopamine release, causing dysphoria and drug withdrawal symptoms [60]. Through these adaptive mechanisms, chronic opioid users develop tolerance, so that in order to obtain the same effect they need to increase the dose. Tolerance develops differently for the various effects of opioids, faster for the analgesic and euphoric effect and slower for the gastrointestinal effect. The pharmacological mechanisms involved in opioid tolerance are complex and yet to be entirely explained, but desensitization and internalization of the μ-opioid receptor seems to be one of the primary causes [61]. Long-term opioid treatment leads to dependence, as such if people stop taking the drug they experience symptoms of withdrawal and hyperalgesia (increased pain sensation); however some people also develop an opioid addiction, characterized by a compulsive urge to take the drug, even though there is no medical requirement [62]. As a consequence, opioid addiction can often lead to drug overdose.

## 4. Prescription Policy, Adherence and Abuse

As pointed out above, to develop more effective strategies to prevent opioid addiction and abuse we have to look beyond biochemical mechanisms and analyze cultural and social settings and mechanisms as well. While the pharmaceutical industry and medical practices certainly fueled the opioid crisis, it needs to be stressed that the key to prevention lies in the patient perspective. The opioid crisis is part of a larger development in the US, in which a number of drugs, including amphetamines, have been both overprescribed [63] and abused [64]. This suggests that the “stimulants” crisis and the opioid crisis can be seen as part of a continuum. Moreover, to understand the patient perspective, we have to look at the cultural underpinnings of prescription policy. Prescription policies and patient adherence to therapeutic regimens do not originate in a void. Rather, there is a cultural background, which shapes them in a multifactorial manner. Some factors are globally present, while others may be locally and culturally specific. It is important here to specify that cultural ramifications of the opioid crisis are in spite of a potentially huge spectrum that is not arbitrary, but that hinges on context-specific factors such as urbanity, levels of industrialization and income structure.

Pharmaceutical biology and biomedicine necessarily look at the patient as a generalizable category which is closely tied to the ideal of medicine as a “colorblind” practice: the physician has to treat the patient with complete disregard of the patient’s ethnicity, gender or social circumstances. This does not mean, however, that specific risk factors related to one of the latter categories can justifiably be neglected. From a humanities perspective, the reference to the “patient” is broken up, in order to explore the cultural, social and economic contexts into which this patient is embedded if not “created”. For the U.S., this cultural embeddedness means that the patient finds himself in a society which is deeply meritocratic, much more so than in, for example, Europe. This means that an individual’s identity is defined by what they can achieve, on a personal, but much more importantly, on an economic scale. The so-called American Dream, where an individual can climb from rags to riches overnight, has been partly shattered, but is still deeply embedded in the self-image of the U.S. The meritocratic ideal implies, however, that all responsibility to create a desirable fate lies with the individual. This is one of the reasons why social welfare has not been very pronounced in the U.S. [65,66].

Interestingly, this has profound consequences for the individual’s attitude to medication. To succeed in this merit-based and highly competitive society, an individual has to constantly perform at their best. This means that they must strive to eliminate all performative insufficiencies and as a consequence, this means one has to get rid of any forms of pain as soon as possible.

The documentary film *Take Your Pills* [67] points to the intersection between economic, cultural and pharmaceutical aspects of the current prescription crisis in the U.S. Even if the film is mostly concerned with the stimulants crisis outlined above, it can also illuminate many of the cultural aspects which underlie the opioid crisis as well. The film sees the pressure of a neoliberal economy as one of the reasons why individuals abuse prescription drugs.

This can be related to general data about the relationship between health, life expectancy and socio-economic status. The concept of “deaths of despair” was introduced by an analysis from 2017, which highlighted the life expectancy difference between the richest and the poorest counties in the U.S. The increased mortality rates among non-Hispanic white Americans is correlated with different socioeconomic factors, including healthcare access and education background, but it is also exacerbated by opioid over-prescription [68].

Moreover, in a neoliberal economy, individuals are required to work in a highly competitive environment, with long working hours and often with little sleep. Moreover, after the global financial crisis from 2008, neoliberal policies and austerity measures accentuated the existing socioeconomic inequalities [69]. There is a psychological dimension to the neoliberal economy. It is implied that the human body is the capital that an individual possesses; the individual has to maintain it at all cost and the responsibility for maintaining this capital lies solely with the individual [70,71]. It can easily be imagined what would happen to a patient after surgery. Because of the pressures of both the economy and the healthcare system, they will tend to go back to work as soon as possible. In order to do so, they may turn to stronger analgesics which promise to work fast, even if this may include severe side effects. According to the documentary *Take Your Pills*, this is true for both professionals and people working in lower-paid positions.

Multiple investigations, including a report from the National Academy of Science, have described the correlation between the social and economic factors and misuse of opioids. On one hand, poverty, hopelessness, despair, poor working conditions and trauma can be associated with increased opioid use. On the other hand, the workplace can be the cause of opioid use [72]. It can be suggested that to counter the misuse of drugs such as OxyContin™, individuals have to be aware of the cultural and economic pressures which may drive them to resort to these drugs. On an economic level, the opioid crisis may in fact be one of the results of a neoliberal economy, with the increasing pressures of the workplace. On a cultural level, it may mean that individuals have internalized the idea that their bodies are their capital, whose performance must be maintained or even enhanced at all cost. Studies have shown that different risk factors (low socioeconomic status, manual labor, geographic regions) can be associated with augmented opioid use, misuse and overdose deaths [72,73,74]. This implies that public discourse and thus the public health discourse about the use of OxyContin™ must also be changed. The widespread use of opioids must hence be seen not as an isolated incident, but as being part of a larger continuum, which is cultural as much as it is biomedical and pharmaceutical. Moreover, this opens up significant new directions for future research, which can compare the situation of the U.S. and Europe. In Europe, social welfare systems are more pronounced than in the US [75]. They could serve as protective factors. This may be one of the reasons, why the opioid crisis has affected the U.S. more strongly than, for instance, Germany and other European countries. One question, which future research will have to consider, is whether with the dismantling of social welfare systems in Europe, economic pressure on individuals will increase, rendering them increasingly vulnerable to addiction. Significantly, researchers from Frankfurt are currently conducting an European Research Council ERC-project, which compares opioid addiction in the U.S. and in Germany [76]. The purpose of the project is to gain a better understanding of the current trends in opioid use and improve the response of the healthcare systems within the European Union to achieve a faster response and better preparedness. Scientists have found that guidelines for long-term use of opioids in chronic non-tumor pain published in 2009 and updated in 2015 in Germany by Deutsche Schmerzgesellschaft have served as a protective factor [77]. In the updated version from 2015, contraindications and indications of opioids were established and clear rules for stopping the therapy were determined. A second update of the German guidelines, which has not been published yet, narrows the indication of opioids and contains an additional chapter regarding the diagnosis and treatment of pharmaceutical opioid misuses. Plus in contrast to the U.S., German Patients and Doctors can access a multimodal pain therapy, for which the costs are covered by the public or private health insurances [77]. So far, researchers agree that while the opioid crisis is threatening Germany, specific factors may prevent addiction from becoming as widespread as in the U.S. [77]. It is hence important to consider both the roots of the opioid crisis and the nationally and culturally specific situation in Europe [77]. Since the opioid crisis has made many victims in the U.S., scientists in Europe, especially in the countries with a high prescription rate, are gathering data regarding prescription of opioids and related overdoses [53,54,55]. Europe is trying to prevent a situation similar to the U.S. and Canada, by improving both information about opioid medication (through ATOME) [52], but also the response of healthcare systems [77]. While the majority of studies have focused on the treatment of opioid addiction [78,79], it is also important to examine social, cultural and economic factors contributing to opioid addiction.

The film *Take Your Pills* shows that the prescription crisis may connect the widespread abuse of stimulants to the opioid crisis. The narratives found in the film can be linked to qualitative analyses which similarly stress the relevance of patient narratives in understanding factors contributing to the opioid crisis [80]. Generally, recent developments in healthcare have emphasized the benefits of including patient narratives. While patient narratives have been present in medical discourse in the form of case studies, the patient narrative adds multiple perspectives that case reports may not contain. This is especially relevant with regard to the cultural, social and economic aspects included in patient narratives [81]. The narratives contained in the film *Take Your Pills* can hence be seen in continuity with qualitative studies investigating narratives of patients with opioid addiction. Recent data show increased trends in amphetamine prescriptions in children and adolescents but also in nonmedical use and emergency department visits [63,64]. This shows that addiction and over-prescription does not only start in adulthood. Additionally, while in 2003 55 countries approved the medication for ADHD, the U.S. is responsible for >92% of the global spending [63]. A large percentage of American children and teenagers are on ADHD medication [63]. One of the doctors interviewed in the film, Dr. Lawrence Diller, goes so far as to speak of the “medicalizing of everyday life.” Public health discourses have to acknowledge that the use of prescription drugs is no longer an exception, a temporary practice, but has become a constant and “normal” aspect of everyday life. This has also been emphasized in an article that discusses how the medicalization of human lives has become normal, in order to correspond to a neoliberal society [82]. An earlier study described how students perceive the illegal use of stimulants. Accordingly, students justify their use for the right reasons and consider them to be without harmful side effects and not different from other aids against fatigue [83]. In the film, several mothers are asked about, why they chose to put their children on ADHD medication. All of them say that they wanted their children to succeed in school, in order to be able to choose a career later on. Social pressure on the individual thus starts incredibly early on. The patterns to which children and adults are subjected in a neoliberal economy today may thus be highly similar; this is also confirmed by the increased trends (344%) in women with private insurance, who filled an ADHD prescription [63].

The film refers to political scientist Wendy Brown. Brown emphasizes that the opioid crisis is located at the intersection between self-enhancement, pain management and cultural and economic pressure. She argues that one way out of the current dilemma, for both the patient and the medical and pharmaceutical industry, is to look at the different factors which contribute to the crisis. Brown states that a starting point would be an individual’s awareness of the pressures, which drive them to take certain medications. Awareness of cultural and economic pressures and the requirements of a neoliberal economy are essential here. At the same time, Brown urges us to look at the “human factor” in this debate. A neoliberal economy, she notes, wants individuals to be perfect, to be infallible. This, however, may run counter to human nature.

What does this mean for pain management? It means, first and foremost, that a person must be able to afford a longer recovery period in order not to have to resort to opioid analgesics on such a large scale. The healthcare system, an individual’s personal healthcare plan and the pressures of a neoliberal economy may work against such an option. In order to counter the current opioid crisis, however, we need to be aware that this crisis is multifactorial: it is made up of medical, pharmaceutical, economic, social and cultural factors together. An in-depth knowledge of the social and cultural factors, which may contribute to the opioid epidemic in turn, may also be important for cognitive behavioral therapy.

As mentioned above, OUD (opioid use disorder) is often associated with alcohol or benzodiazepine use as well as psychiatric disorders (depression). As we can see, there is no easy solution to this problem. As such, medication-assisted treatment (MAT) has been developed to prevent relapse and improve patient survival. MAT combines FDA-approved medicine to treat OUD (methadone, naloxone and buprenorphine) with counseling and behavioral therapies. Methadone is a long-acting opioid agonist, which suppresses withdrawal and craving symptoms and reduces the effects of illicit opioids (heroin). Methadone is used as both detoxification and maintenance medication in the treatment of OUD [1,84]. Buprenorphine is a partial μ-opioid receptor agonist, which similar to methadone reduces withdrawal symptoms and opioid craving. Buprenorphine formulations are available either in monotherapy or in combination with naloxone [1,84]. Naltrexone is a competitive antagonist of opioid receptors which blocks the effects of opioids (oxycodone). Because it produces severe withdrawal symptoms, it is not indicated during the detoxification phase, but rather for a maintenance medication [1]. Multiple studies proved the efficiency of methadone maintenance therapy and psychosocial treatment (CBT, supportive counseling), as patients showed better improvement and overall outcome at a 12-month follow up. Additionally, similar results were obtained for patients receiving naltrexone or buprenorphine maintenance therapy in combination with psychosocial treatment. These patients demonstrated increased treatment attendance and therapy compliance [1]. The ongoing COVID-19 crisis, however, demonstrates how fragile these structures are. Social deprivation, the shut-down of institutions and methadone programs, the aggravation of precarious life situations and poverty shed a blinding light on the underpinnings of the opioid crisis.

## 5. Why It Is Important to Understand the Patient’s Perspective

In the context of public health and public understanding of the opioid crisis, it is especially interesting that the CDC website hosts an entire collection of personal narratives about opioid addiction [85]. These narratives are written either by former patients or by their relatives. For an understanding of the ongoing threat caused by opioid use and abuse to human health, this collection of first-person narratives is informative both from an ethical and a methodological perspective. From an ethical perspective, personal accounts of addiction may serve the purpose of providing access to an authentic re-evaluation, weighing individual behavior in the form of first-order wish-like instant satisfaction (removing pain, being shielded from life, feeling better) against higher-order wishes based on the reflection of more fundamental values (managing pain, leading a life, being well). As the national Centers for Disease Control, the CDC not only gathers statistics and reports on problematic developments in population health but should also help to counteract such developments. The fact that the CDC should include on its website not only statistics, but also patient narratives, supports the argument that approaches to combating the opioid epidemic should be multidisciplinary and may stress patient narratives as one resource for understanding individual, social and cultural factors. At the same time, it can be argued that the patient narratives provided on the CDC website may be showcases of ideal, desirable developments, rather than in-depth accounts of the complexities which make up opioid addiction. This is why the documentary *Take Your Pills* is an important example of how patient narratives are embedded into a much more complex account of social, environmental, rural or urban settings, and socioeconomic disenfranchisement. The rich content of such narratives can and should be used to reframe preventive strategies as knowledge-based, evidence-driven and socially and culturally embedded in an authentic way at the same time. Turning groups at risk into communities that need to be addressed in ways specific and relevant to their settings is one of the major challenges for healthcare in the ongoing opioid crisis. This corresponds to qualitative studies, which stress the need for including narratives in research about the opioid crisis. Such studies have addressed the relevance of narrative understanding for doctor–patient relationships. For instance, an analysis suggested that the doctor and patient are equally in pain: for the patient, the situation is about pain management; but for the physician, “pain” may involve understanding the complexity of the patient’s somatic, social, cultural and economic condition and the impetus to help the patient while precluding addition [86].

On a methodological level, for both humanities research and research at the intersection between pharmaceutical biology and the humanities, this raises important questions. From a humanities perspective, personal narratives can themselves be seen as a form of knowledge about human life; they provide what Ottmar Ette has called a form of *Lebenswissen* (life knowledge) [87,88]. From a humanities perspective, analyzing patient narratives may hence be key in contributing to the knowledge base which is needed to understand the opioid epidemic. Studies have shown that these narratives can increasingly be found on social media [80]. They are also contained on health-related websites such as the Colorado Office of Behavioral Health [89].

These life narratives, in turn, can help awareness of a given development (here, the perils of opioid addiction) for the wider public. It is essential here that these are not fictional stories; they can be seen as patient narratives. As such patient narratives, they make people aware of the concrete danger of becoming addicted to opioids. Moreover, because people can relate to the stories of the people on the website, they may become aware that this could easily happen to them as well. It is important to note here that the people whose stories are being told on the website are referred to by their first names. This has the effect that readers will relate to them on a personal level and will become aware that this could easily become their own story. Recent studies have investigated the role of narratives both for treating addiction [90] and for increasing social awareness about the risk factors leading to addiction. These studies stress that patient narratives contain turning points in individual narratives: by telling their stories, patients become aware at what point they were becoming more susceptible to addiction [90]. Conversely, readers of patient narratives such as the ones hosted by the CDC may be enabled to assess, whether they are prone to addiction. To the extent that they recognize parallels between their own lives and the life course described in patient narratives, they may realize that they, too, may be at risk.

As seen on the CDC page, patients who narrate the story of their addiction with opioids come from all walks of life: they are college or high school students or financial advisers; they are white, African American, or Hispanic [85]. Through this cross section of society, it becomes clear that the opioid crisis can affect anyone, despite their social status or professional background. Through their selection of stories, the CDC thus targets common misconceptions about the people that become addicted to opioids.

The patient narratives contained on the CDC website and in the film *Take Your Pills* are in line with recent studies that stress the role of “storytelling” in addiction prevention [91]. These studies stress that addiction can be seen not only as a health problem, but also as a social problem [91]. This view can be applied to the use of narratives in the opioid epidemic and the use of narratives by the CDC and the documentary. Patient narratives reveal underlying patterns about consumption patterns [91] and social conditions, which may lead to such patterns. As these studies show, a biological perspective on opioid addiction and a narrative approach can be mutually complementary [92].

Patient narratives can then be related to prescription policies, both in the U.S. and in other parts of the globe. If pain management is recognized by the WHO as a fundamental human right, it may nevertheless be important for physicians to debate for what conditions certain opioids are prescribed. It could thus be argued that the narratives provided on the CDC website shy away from addressing a much more fundamental problem: the relationship between social inequality, physical labor, chronic pain and opioid addiction. At this juncture, the usual narratives of coping can be misleading. Opioid addiction does not happen to people regardless of specific personal, social or economic circumstances. We suggest here, on the other hand, that in order to adequately target the opioid crisis and the factors leading up to it, it is important to take social inequality into account particularly on the levels of assessment, assurance, and policy making. This has far-reaching implications for methodology. Research on patient narratives must fruitfully intersect not only with a detailed understanding of the scientific mechanisms leading to an opioid addiction, but they must also be fed into higher-order discussions of the general parameters of the healthcare system. In particular, one of the most central problems of the healthcare system must be to take into account social inequality and unjust access to health [93]. For an understanding and tackling of the opioid epidemic, this implies that socially and economically disenfranchised groups may be seen as particularly vulnerable to opioid addiction. Thus, an integrative analysis of opioid addiction must take into account the pressures of the workplace and the economic system as much as it must consider individual factors in the framework of adequate medical treatment and care.

The above-mentioned shortcomings notwithstanding, however, these personal narratives point to a number of factors which can be informative for combating the opioid crisis. The narratives link medical and pharmaceutical practice to a cultural discussion about opioids. The biggest threat that these personal narratives describe is this one: The prescription of opioid-based analgesics has been “normalized” in US culture. Physicians may easily prescribe them, even in instances when this may not be absolutely necessary or even when there is a contraindication if anamnestic knowledge is sufficiently generated. Patients, on the other hand, may conceive of these medications as “harmless”, and may not be aware of the danger of addiction. In countering the current opioid crisis, life sciences and humanities can inform public health about the multiple factors which have led to this crisis. In view of these considerations, it is important to consider opioid addiction in a global context. Knowledge of different languages apart from English is needed in order to analyze patient narratives not only from the U.S. and the UK, but also from many European countries. Patient narratives may contain important information concerning patient attitudes [94]. Socio-economic factors, culture-specific attitudes and different prescription policies must be viewed from a comparative perspective.

## 6. Conclusions

This paper has suggested that, in order to understand the current opioid crisis, a multifactorial approach is necessary which links approaches from pharmaceutical biology to a cultural and social perspective. To counteract the present crisis, it is important to consider the different perspectives of physicians, pharmaceutical companies, patients and their relatives, and also society at large. Established countermeasures turned out to be quick fixes rather than game changers. This holds particularly true in the light of the collision of the COVID-19 and the opioid epidemics [95]. The opioid epidemic has evolved rapidly during the last decades. According to the data gathered by Substance Abuse and Mental Health Services Administration (SAMHSA), in 2016 over 63,000 people died of drug overdose in the U.S., most of them involving a synthetic opioid or heroin, and 2.1 million people suffered from an opioid use disorder (OUD) [96]. The consequences of OUD include contracting hepatitis B and C virus, human immunodeficiency virus (HIV) or overdose, and on a psychosocial level financial and legal problems and unemployment [84].

For a widespread misuse of a given substance to occur, society had to normalize the use of this substance and hence make it culturally acceptable. As the opioid crisis shows, this normalization can have disastrous consequences [96]. This paper has considered the role of the CDC on a number of levels. As the national health protection agency, the aim of the CDC is to “increase health security” [85]. Regarding the growing number of people with an opioid addiction, the CDC has attempted to raise awareness about the cultural, social and personal mechanisms which can cause the addiction. By providing an overview of general policies and by including patient narratives, the CDC website could be considered a tool for the wider public and for practitioners. This suggests that public health institutions have an important, if not essential, role in fighting the opioid epidemic and need to adjust their strategies according to the fact that dealing with social inequalities, precarious lives and unjust access to health is critical for dealing with the opioid crisis.

This paper has suggested that this crisis is a pharmaceutical and medical crisis as well as a cultural one. Individuals may be prone to misuse opioids to keep up with the pressure of the workplace in a neoliberal economy and in a highly competitive education system. As patient narratives show, opioid addiction can start early on in an individual’s life. At the same time, physicians may have been too quick to prescribe opioids or may have failed to alert their patients to the dangers of addiction. The pharmaceutical industry, too, may have stressed only the advantages of opioid medication. In this paper, we have argued that a multidisciplinary approach which combines pharmaceutical biology with a cultural and special perspective can alert all the actors involved in the opioid crisis to the different factors involved in bringing about addiction. With such a multifactorial approach, public health strategies can be reshaped to battle the opioid crisis more efficiently. On both a therapeutic and a preventive level, one solution to the opioid crisis may thus lie in raising awareness. Education and awareness-raising may be one of the most effective ways of combating the ubiquitous use of opioids.
